# Phosphoregulation of the dimerization and functions of end-binding protein 1

**DOI:** 10.1007/s13238-014-0081-9

**Published:** 2014-07-23

**Authors:** Jie Chen, Youguang Luo, Lixin Li, Jie Ran, Xincheng Wang, Siqi Gao, Min Liu, Dengwen Li, Wenqing Shui, Jun Zhou

**Affiliations:** 1 State Key Laboratory of Medicinal Chemical Biology, College of Life Sciences, Nankai University, Tianjin, 300071 China; 2 High-Throughput Molecular Drug Discovery Center, Tianjin Joint Academy of Biotechnology and Medicine, Tianjin, 300457 China

**Dear Editor,**

End-binding protein 1 (EB1), a member of microtubule plus-end tracking proteins (+TIPs), plays an important role in the regulation of microtubule dynamics and has been implicated in cancer development (Dong et al., 2010; Gouveia and Akhmanova, 2010; Wang et al., 2005). However, it remains poorly understood how EB1 functions are regulated by phosphorylation in mammalian cells. To map the phosphorylation pattern of EB1, we overexpressed GST-EB1 in HeLa cells and enriched GST-EB1 from cell lysates by GST pulldown. The pulldown preparations showed strong serine, threonine, and tyrosine phosphorylation signals in the immunoblots (Fig. S1A). GST-EB1 purified from cells was clearly visualized on the gel prior to in-gel digestion (Fig. S1B).

Nanoscale liquid chromatography coupled to tandem mass spectrometry analysis of the EB1 peptides identified 11 new phosphorylation sites, among which S27, T33, and Y71 are located in the calponin homology (CH) domain, T154, S155, S156, S157, S165, and T166 in the linker region, and T206 and Y217 in the end binding homolog (EBH) domain (Fig. S1C and S1D). It is noteworthy that for the consecutive linker-region residues T154, S155, S156, and S157 (TSSS), and S165 and T166 (ST), the phosphorylation sites could not be unambiguously assigned based on the mass spectrometry profiles (Figs. S1D and S2), yet they were all likely to be phosphorylated with certain stoichiometry. Therefore, we treated TSSS or ST as one phosphorylation motif and mutated all the residues in the motif in subsequent functional assays. According to the crystal structures of the CH and EBH domains available in the protein data bank, we analyzed the localization of five identified phosphorylation sites in the three-dimensional structures. By molecular modeling, we found that all the five phosphosites present in the CH and EBH domains were exposed to the surface of EB1 (Fig. S1E).

To explore the functional roles of EB1 phosphorylation, we generated a panel of phospho-deficient (mutation of serine and threonine to alanines and mutation of tyrosine to phenylalanine) and phospho-mimic (mutation of serine, threonine, and tyrosine to aspartic acids) mutants. Immunoblot analysis with antibodies against phosphorylated serine and threonine did not show any dramatic changes in EB1 phosphorylation level for the S27A (mutation of serine 27 to alanine), T33A (mutation of threonine 33 to alanine), TSSSAAAA (mutation of threonine 154, serine 155, serine 156, and serine 157 to alanines), STAA (mutation of serine 165 and threonine 166 to alanines), and T206A mutants (mutation of threonine 206 to alanine) (Fig. S3A). Considering that in total five serines and four threonines were identified to be phosphorylated, it was conceivable that the overall phosphorylation level of EB1 was not significantly altered by single-site or single-motif mutations. By contrast, the phosphorylation of Y71 and Y217 contributed significantly to the overall tyrosine phosphorylation of EB1, as both of the phospho-deficient mutants showed dramatically reduced signals in the immunoblot, with antibodies against phosphorylated tyrosine (Fig. S3B). We then overexpressed wild-type or mutant EB1 to investigate whether the phosphorylation at specific sites affects EB1 interaction with microtubules/tubulin. It turned out that none of the EB1 mutants changed its interaction with α-tubulin in the GST pulldown assays (Fig. S3C–E). Immunofluorescence microscopy further revealed that all the mutants of EB1 were located at the plus end of microtubules in a pattern similar to wild-type EB1 (Fig. S3F).

To analyze the effect of EB1 phosphorylation on microtubule dynamics, we overexpressed GFP-EB1 wild-type and mutants and took serial images by time-lapse microscopy. We then used the PlusTipTracker software to analyze the dynamics of microtubules (Matov et al., 2010). According to the mean growth speed (15 μm/min) and mean growth time (9 s) of wild-type EB1, we divided the microtubule population into four groups (Fig. 1A and 1B). We considered cells with a high percentage of fast-growth and long-lived microtubules highly dynamic. Both T33A and T33D decreased the percentage of fast-growth and long-lived microtubules, and the growth speed and growth length decreased significantly as compared to wild-type EB1 (Fig. 1C–E), indicating the importance of T33 for EB1 to regulate microtubule dynamics. As for Y71, TSSS, and T206, we found that all the phospho-deficient mutants decreased the dynamics, growth speed, and growth length of microtubules, whereas the phospho-mimic mutants either maintained or promoted the dynamics, growth speed, and growth length of microtubules (Fig. 1C–E). As for S27 and ST, although the phospho-deficient mutants did not significantly decrease the dynamics, growth speed, or growth length of microtubules, S27D slightly promoted the growth speed and growth length of microtubules and STDD promoted the dynamics, growth speed, and growth length of microtubules (Fig. 1C–E). These results suggested that the phosphorylation of Y71, TSSS, and T206 is needed for EB1 to control microtubule dynamics. In addition, by time-lapse microscopy, we found that the Y217F and Y217D mutants had significantly decreased ability to track microtubule plus ends (Fig. 1F and 1G).

**Figure 1:**
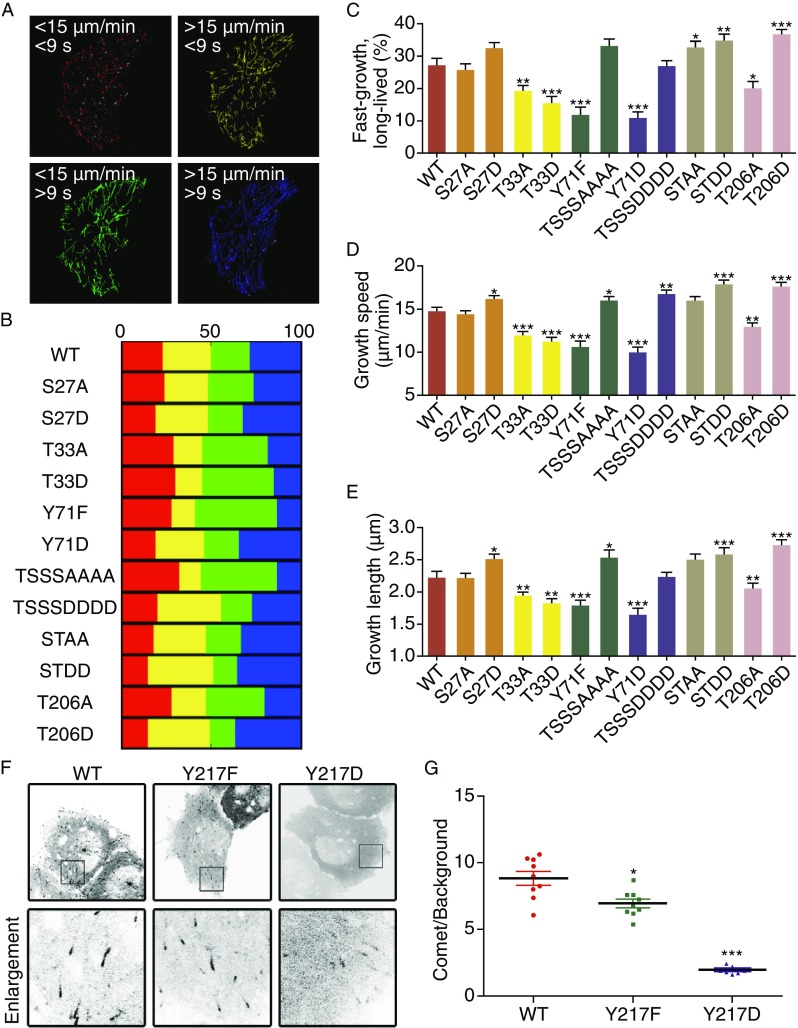
**EB1 phosphorylation at different sites modulates microtubule dynamics to different extents**. (A–C) HeLa cells were transfected with GFP-EB1 or various mutants, and time-lapse images of GFP-EB1 were taken by confocal microscopy at 2-second intervals. The images were analyzed with the PlusTipTracker software and the Quadrant Scatter Plot tool. Microtubules were classified into four subpopulations based on the mean growth speed (15 μm/min) and mean growth time (9 s) of GFP-EB1, and representative images were shown in (A). The partitioning of the four subpopulations of microtubules was shown in (B), and the percentage of fast-growth and long-lived microtubules was shown in (C). (D) Mean growth speed of microtubules. (E) Mean growth length of microtubules. (F) Cells were transfected with GFP-EB1 wild-type or the Y217F and Y217D mutants, and time-lapse images of GFP-EB1 were taken by confocal microscopy at 2-second intervals. (G) Experiments were performed as in (F), and the relative fluorescence intensity of the GFP-EB1 comets was measured. *P < 0.05, **P < 0.01, ***P < 0.001. Error bars indicate SEM

Because EB1 interacts with +TIPs through its carboxyl terminus, we chose T206, Y217, and another site near the carboxyl terminus, ST, for experiments investigating EB1 interaction with +TIPs. We selected adenomatous polyposis coli (APC) and mitotic centromere-associated kinesin (MCAK) as examples for SxIP motif-containing proteins (Honnappa et al., 2009). Y217D, but not T206D or STDD, abrogated the interaction of EB1 with MCAK and APC (Fig. 2A). Unexpectedly, Y217F also abrogated the interaction of EB1 with MCAK and APC (Fig. 2A). We then analyzed the three-dimensional structure of the carboxyl terminus of EB1 and found that Y217 could form a hydrogen bond with the proline residue of the SxIP motif; by contrast, Y217F and Y217D failed to form a hydrogen bond with the proline residue in SxIP (Fig. 2B). This might explain the result that Y217F decreased the ability of EB1 to interact with MCAK and APC. It was reported that Y217A barely interacted with microtubule actin cross-linking factor 2 (MACF2), but Y217F could interact with MACF2 slightly (Slep et al., 2005). Considering that phosphorylation near the SxIP motif can abrogate the interaction between EB1 and +TIPs (Honnappa et al., 2009), we speculated that amino acids with negative charge (D or E) near the SxIP motif might be responsible for the different abilities of EB1 to interact with different +TIPs (Fig. 2C).

**Figure 2:**
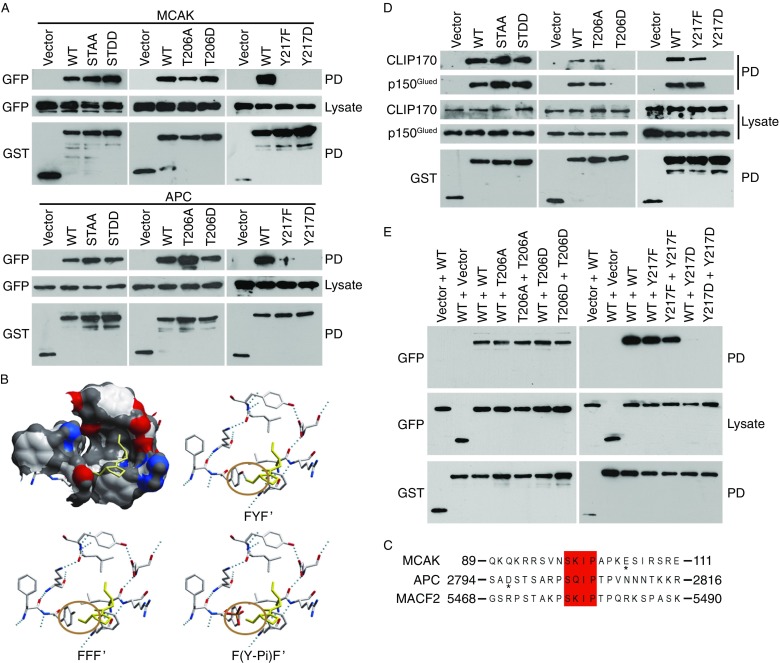
**EB1 phosphorylation at Y217 regulates its interaction with other +TIPs as well as its dimerization**. (A) Cells were transfected with GFP-MCAK or GST-APC, together with GST, GST-EB1 wild-type, or various mutants. GST pulldown and immunoblotting were then performed with the indicated antibodies to analyze the interaction of EB1 with MCAK or APC. (B) Schematic model showing the interaction of the hydrophobic cavity of EB1 with the SxIP motif. The yellow peptide in the upper-left model indicates the IP residues in the SxIP motif. A hydrogen bond is formed between Y217 in the hydrophobic cavity of EB1 and the P residue in the SxIP motif. When Y217 is replaced by F or modified by phosphorylation, the hydrogen bond is disrupted. (C) Alignment of the SxIP motifs and the adjacent sequences of MCAK, APC, and MACF2. The negatively charged residues are indicated with asterisks. (D) Cells were transfected with GST, GST-EB1 wild-type, or various mutants. GST pulldown and immunoblotting were then performed with the indicated antibodies to analyze the interaction of EB1 with CLIP170 and p150^Glued^. (E) Cells were transfected with GST, GST-EB1 wild-type, or the indicated mutants, together with GFP, GFP-EB1 wild-type, or the mutants. GST pulldown and immunoblotting were then performed to examine EB1 dimerization

Among the +TIPs, cytoplasmic linker protein of 170 kDa (CLIP170) and p150Glued are known to interact with EB1 through cytoskeleton-associated protein glycine-rich (CAP-Gly) domains (Honnappa et al., 2009). As described above, we chose the T206, Y217, and ST mutants of EB1 for experiments investigating the interaction of EB1 with CLIP170 and p150Glued. The STDD mutant did not obviously affect their interactions (Fig. 2D), but T206D and Y217D could abrogate the interaction of EB1 with CLIP170 and p150Glued (Fig. 2D). Because EB1 dimerization is required for its interaction with p150Glued (Honnappa et al., 2006), we sought to examine whether T206D and Y217D affect EB1 dimerization. We found that Y217D, but not T206D, destroyed EB1 dimerization (Fig. 2E). This result suggested that the phosphorylation of EB1 at Y217 might take negative charge into the hydrophobic cavity formed by two EB1 monomers, leading to the collapse of the hydrophobic cavity and disruption of EB1 dimerization.

Free EB1 dimers are known as elongated and globally asymmetric molecules, of which the EBH domains form a 9-nanometer rod and the two CH domains form an 8-nanometer dumbbell structure (Buey et al., 2011). EB1 dimers are much more compact in structure compared with EB1 monomers, because the relative positions of the CH and EBH domains of EB1 monomers are variable (Fig. S4A and S4B). Since free EB1 dimers are asymmetric, the two CH domains in an EB1 dimer are different (Fig. S4A). It is also known that the CH domain of EB1 binds to the corner of four tubulin dimers through its amino-terminal CH domain (Maurer et al., 2012). It is possible that EB1 dimers bind to microtubules in a certain manner. One of the two CH domains in the EB1 dimer might bind to the corner of four tubulin dimers, whereas the other CH domain just stays in the next corner of the four tubulin dimers and does not participate in the interaction between EB1 dimer and microtubules (Fig. S4A). For EB1 monomers, their variable structure renders their binding to microtubules in chaos due to steric hindrance, and less EB1 monomers are located at the limited binding sites of microtubules compared with EB1 dimers (Fig. S4B). In this model, EB1 dimerization is necessary for maintaining the proper level of EB1 tracking the growing microtubules.

Considering that the concentration of EB1 is hundreds of nanomoles in cells and that the concentration of EB1 needed for dimerization is lower than 1 nanomole, the newly translated EB1 monomers are obligatory to form dimers immediately and it is quite hard for EB1 dimers to dissociate into monomers (Sen et al., 2013). EB1 dimers can interact with other +TIPs and recruit them to the plus end of microtubules. Since free EB1 and microtubule-bound EB1 are obligatory to form dimers, free EB1 dimers can compete with the microtubule-bound EB1 dimers for binding to +TIPs. Because +TIPs function at the plus end of microtubules, free EB1 dimers binding to other +TIPs is an uneconomic event. This may provide an explanation for the finding that EB1 monomers are unable to bind to other +TIPs such as MCAK, APC, CLIP170, and p150^Glued^. In this scenario, it is possible that at the plus end of microtubules, EB1 dimers bind to other +TIPs, whereas in the cytoplasm most of EB1 is phosphorylated at Y217 to stay as monomers, which could not bind to other +TIPs (Fig. S4C). Further experiments are warranted to investigate this possibility.

Taken together, we identify by mass spectrometry a number of phosphorylation sites in EB1 and find that EB1 phosphorylation does not significantly affect its interaction with microtubules, but modulates microtubule dynamics to different degrees. In addition, we demonstrate that EB1 phosphorylation at Y217 regulates its interaction with other +TIPs and the equilibrium of EB1 between monomer and dimer forms. These findings suggest that the functions of EB1 in the regulation of microtubule dynamics and recruitment of other +TIPs, as well as the dimerization of EB1, undergo exquisite control by phosphorylation.

## Electronic supplementary material

Below is the link to the electronic supplementary material.

Supplementary material 1 (PDF 479 kb)

## References

[CR1] Buey RM, Mohan R, Leslie K, Walzthoeni T, Missimer JH, Menzel A, Bjelic S, Bargsten K, Grigoriev I, Smal I (2011). Insights into EB1 structure and the role of its C-terminal domain for discriminating microtubule tips from the lattice. Mol Biol Cell.

[CR2] Dong X, Liu F, Sun L, Liu M, Li D, Su D, Zhu Z, Dong JT, Fu L, Zhou J (2010). Oncogenic function of microtubule end-binding protein 1 in breast cancer. J Pathol.

[CR3] Gouveia SM, Akhmanova A (2010). Cell and molecular biology of microtubule plus end tracking proteins: end binding proteins and their partners. Int Rev Cell Mol Biol.

[CR4] Honnappa S, Okhrimenko O, Jaussi R, Jawhari H, Jelesarov I, Winkler FK, Steinmetz MO (2006). Key interaction modes of dynamic +TIP networks. Mol Cell.

[CR5] Honnappa S, Gouveia SM, Weisbrich A, Damberger FF, Bhavesh NS, Jawhari H, Grigoriev I, van Rijssel FJ, Buey RM, Lawera A (2009). An EB1-binding motif acts as a microtubule tip localization signal. Cell.

[CR6] Matov A, Applegate K, Kumar P, Thoma C, Krek W, Danuser G, Wittmann T (2010). Analysis of microtubule dynamic instability using a plus-end growth marker. Nat Methods.

[CR7] Maurer SP, Fourniol FJ, Bohner G, Moores CA, Surrey T (2012). EBs recognize a nucleotide-dependent structural cap at growing microtubule ends. Cell.

[CR8] Sen I, Veprintsev D, Akhmanova A, Steinmetz MO (2013). End binding proteins are obligatory dimers. PLoS One.

[CR9] Slep KC, Rogers SL, Elliott SL, Ohkura H, Kolodziej PA, Vale RD (2005). Structural determinants for EB1-mediated recruitment of APC and spectraplakins to the microtubule plus end. J Cell Biol.

[CR10] Wang Y, Zhou X, Zhu H, Liu S, Zhou C, Zhang G, Xue L, Lu N, Quan L, Bai J (2005). Overexpression of EB1 in human esophageal squamous cell carcinoma (ESCC) may promote cellular growth by activating beta-catenin/TCF pathway. Oncogene.

