# Supplementary material for: Phosphoregulation of the dimerization and functions of end-binding protein 1
Source: Protein Cell. 2014 Jul 23;5(10):795–9. doi: 10.1007/s13238-014-0081-9 (PMC4180461; doi:10.1007/s13238-014-0081-9)
Supplement: Supplementary file 1 — Supplementary material 1 (PDF 479 kb) [file 13238_2014_81_MOESM1_ESM.pdf]

Phosphoregulation of the dimerization and functions of end-binding protein 1

Jie Chen, Youguang Luo, Lixin Li, Jie Ran, Xincheng Wang, Siqi Gao, Min Liu, Dengwen Li, Wenqing Shui, and Jun Zhou

Supplemental Materials

Materials and Methods

Materials

Antibodies against GST, GFP, and α-tubulin (Sigma-Aldrich), phosphorylated serine and threonine (Qiagen), phosphorylated tyrosine (Millipore), and CLIP170 and $p150^{Glued}$ (Santa Cruz Biotechnology) were obtained from the indicated sources. Horseradish peroxidase-conjugated secondary antibodies were obtained from Amersham Biosciences, and fluorescein-conjugated secondary antibodies were from Jackson ImmunoResearch Laboratories. Mammalian expression plasmids for GST-EB1 and GFP-EB1 were constructed by PCR using the pEBG and pEGFPN1 vectors, respectively, and various mutants of EB1 were generated by site-directed mutagenesis. GFP-APC and GFP-MCAK were constructed by using the pEGFPC1 vector. HeLa cells were cultured in the DMEM medium supplemented with 10% FBS at 37°C in a humidified atmosphere with 5% CO_2. Plasmids were transfected with the polyethyleneimine reagent (Sigma-Aldrich).

GST pulldown and immunoblotting

Cell lysates were incubated with glutathione agarose beads (Promega) at 4°C for 2 hours. The beads were washed extensively and boiled in the SDS loading buffer. The proteins were resolved by SDS-PAGE and transferred onto polyvinylidene difluoride membranes (Millipore). The membranes were blocked in Tris-buffered saline containing 0.2% Tween 20 and 5% fat-free dry milk and incubated first with primary antibodies and then with horseradish peroxidase-conjugated secondary antibodies. Specific proteins were visualized with enhanced chemiluminescence detection reagent (Millipore).

In-gel digestion and mass spectrometric analysis

GST-EB1 was purified from cells with glutathione agarose beads, resolved by SDS-PAGE, and stained with Coomassie blue. The GST-EB1 band was then subjected to standard in-gel digestion. Eluting peptides were loaded onto a Waters Symmetry C18 trapping column (300 μm i.d. 1 cm length) using the Waters NanoAcquity UPLC system, and separated by a linear gradient from 2% to 35% B over 40 min (A = 0.1% FA in H_2O, B = 0.1% FA in ACN) at 300 nl/min through a 100 μm i.d. 10 cm column packed with 1.7 μm BEH C18 material (Waters). The Waters Synapt Q-IM-TOF G1 mass spectrometer was operated in high-definition MS^E mode, and the data were processed with ProteinLynx Global Server (PLGS v2.4, Waters) to reconstruct MS/MS spectra by combining all masses with a similar retention time. MS/MS

spectra were searched against UniProt human sequence database using PLGS with the following parameters: peak width, 0.3 min; MS and MS/MS tolerance, automatic (typical mass error <15 ppm for MS and <30 ppm for MS/MS); trypsin missed cleavages, 1; fixed modification, carbamidomethylation; variable modifications, Met oxidation and phosphorylation of Ser, Thr, or Tyr; at least 5 independent product ions assigned for a peptide identification. The false positive rate was <1% at both the protein and peptide levels. Phosphosite identification had to meet the following criteria: (1) phosphopeptides were identified with a confidence >95% and a PLGS peptide score >6; (2) the mass error of the peptide precursor was below 10 ppm; (3) the MS/MS spectrum was manually inspected to confirm neutral loss and specific fragment ions critical for assigning the modification sites; and (4) phosphorylation sites were assigned consistently in three biological replicates.

Molecular modeling

The crystal structures of EB1 were from the protein data bank (PDB #2QJZ and PDB #1WU9). The Molsoft ICM software was used to analyze the locations of the five phosphorylated residues in the CH and EBH domains of EB1 and the binding of the SxIP motif to the hydrophobic cavity of EB1.

Immunofluorescence microscopy

Cells grown on glass coverslips were fixed with methanol for 3 minutes at -20°C. Cells were blocked with 2% bovine serum albumin in phosphate-buffered saline and incubated in succession with primary and secondary antibodies. Cells were observed with a fluorescence microscope (Carl Zeiss).

Live-cell imaging and analysis

HeLa cells transfected with various GFP-EB1 plasmids were examined with a confocal microscope (Leica) at 37°C with 5% CO_2, and time-lapse images were taken every 2 seconds . The dynamics of microtubules were analyzed by the PlusTipTracker software. The Quadrant Scatter Plot tool, a part of the PlusTipTracker package, was used to determine the relationship between the growth rate and growth lifetime and to divide microtubules into four subpopulations based on the deviations from the mean growth speed and growth lifetime of wild-type EB1. For each subpopulation, 20 cells were analyzed by the PlusTipTracker software and the Quadrant Scatter Plot tool. The percentage of fast-growth and long-lived microtubules, the growth speed of microtubules, and the growth length of microtubules were then calculated with the GraphPad Prism software. To analyze the relative fluorescence intensity of GFP-EB1 comet, the fluorescence intensities of the comet and the background were measured with the ImageJ software and the comet/background ratio was then calculated.

Legends of Supplemental Figures:

Figure S1

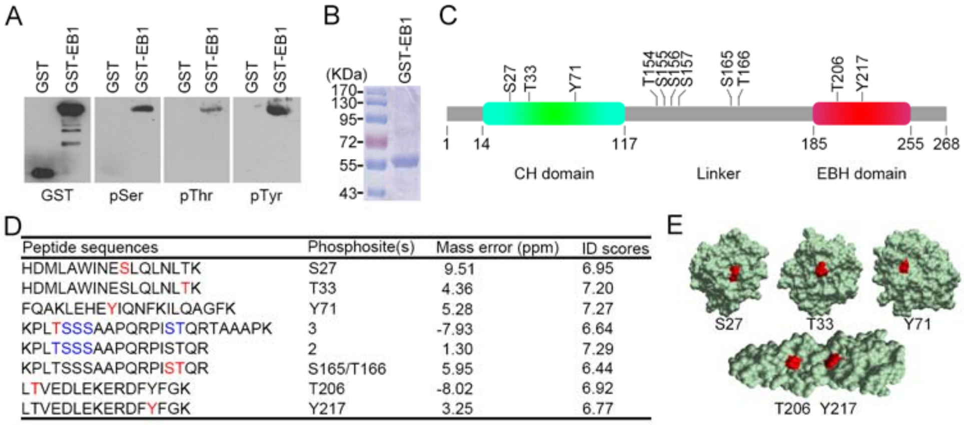

Peptide sequences	Phosphosite(s)	Mass error (ppm)	ID scores
HDMLAWINESLQLNLTK	S27	9.51	6.95
HDMLAWINESLQLNLTK	T33	4.36	7.20
FQAKLEHEYIQNFKILQAGFK	Y71	5.28	7.27
KPLTSSSAAPQRPISTQRTAAAPK	3	-7.93	6.64
KPLTSSSAAPQRPISTQR	2	1.30	7.29
KPLTSSSAAPQRPISTQR	S165/T166	5.95	6.44
LTVEDLEKERDFYFGK	T206	-8.02	6.92
LTVEDLEKERDFYFGK	Y217	3.25	6.77

Figure S1. Mass spectrometry reveals EB1 phosphorylation at multiple sites in mammalian cells. (A) Cells were transfected with GST or GST-EB1, and the pulldown preparations of cell lysates were immunblotted with antibodies against GST or phosphorylated serine, threonine, or tyrosine. (B) Coomassie blue-stained gel of GST-EB1 purified from HeLa cells. (C) Schematic representation of EB1 phosphorylation sites identified in this study. The phosphorylation sites are located in the CH and EBH domains and the linker region. (D) Phosphopeptides of EB1 identified by mass spectrometric analysis. The residues in red are confidently assigned phosphosites, whereas the residues in blue indicate that the phosphosites are not unambiguously assigned and all the clustered residues in blue are likely to undergo phosphorylation. (E) The locations of the five phosphorylated residues in the CH and EBH domains are highlighted in the crystal structures of the CH and EBH domains of EB1.

Figure S2

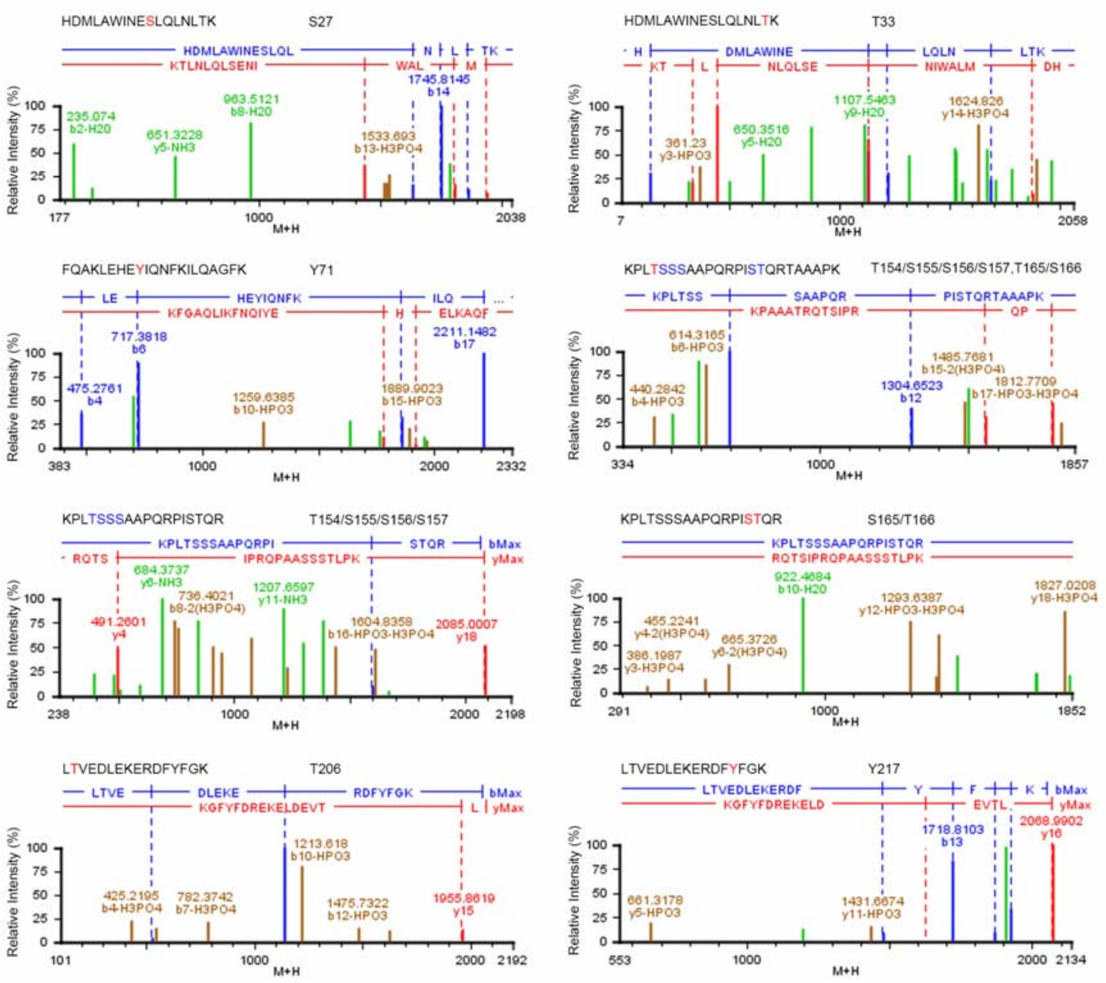

Figure S2. Mass spectrometric profiles of GST-EB1 phosphopeptides.

Figure S3

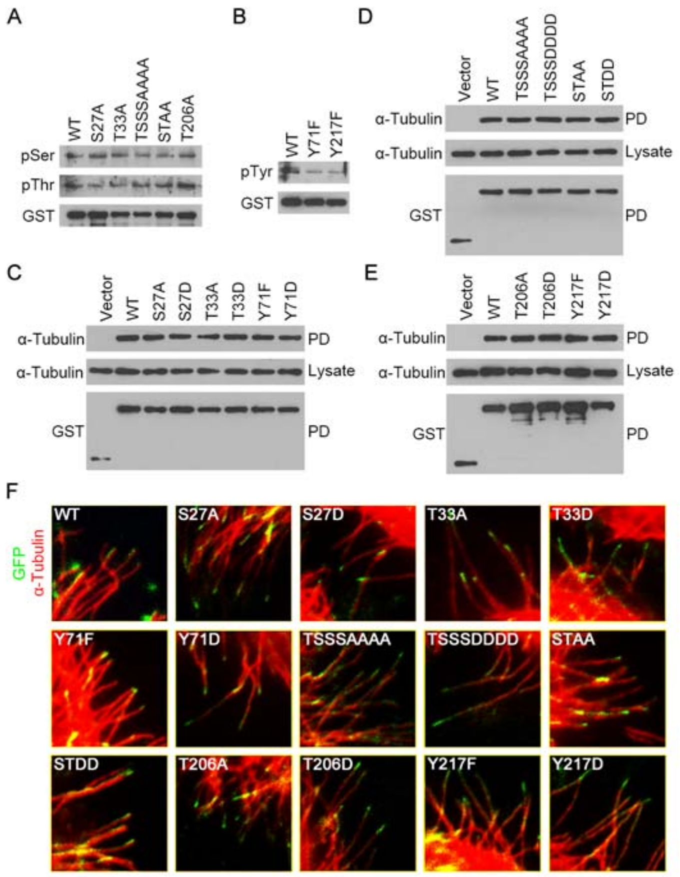

Figure S3. EB1 phosphorylation does not significantly affect its interaction with microtubules or tubulin. (A) Cells were transfected with GST-EB1 wild-type (WT) or mutants (S27A, T33A, TSSSAAAA, STAA, T206A), and the cell lysates were immunoblotted with antibodies against pSer, pThr, or GST. TSSSAAAA indicates that threonine 154, serine 155, serine 156, and serine 157 are mutated to alanines. STAA indicates that serine 165 and threonine 166 are mutated to alanines. (B) Cells were transfected with GST-EB1 wild-type or mutants (Y71F and Y217F), and the cell lysates were immunoblotted with antibodies against pTyr or GST. (C-E) Cells were transfected with GST, GST-EB1 wild-type, or mutants, and the pulldown preparations or cell lysates were immuoblotted with antibodies against α-tubulin or GST. (F) Cells were transfected with GFP-EB1 wild-type or mutants (green), immunostained with α-tubulin antibody to visualize microtubules (red), and then examined by fluorescence microscopy.

Figure S4

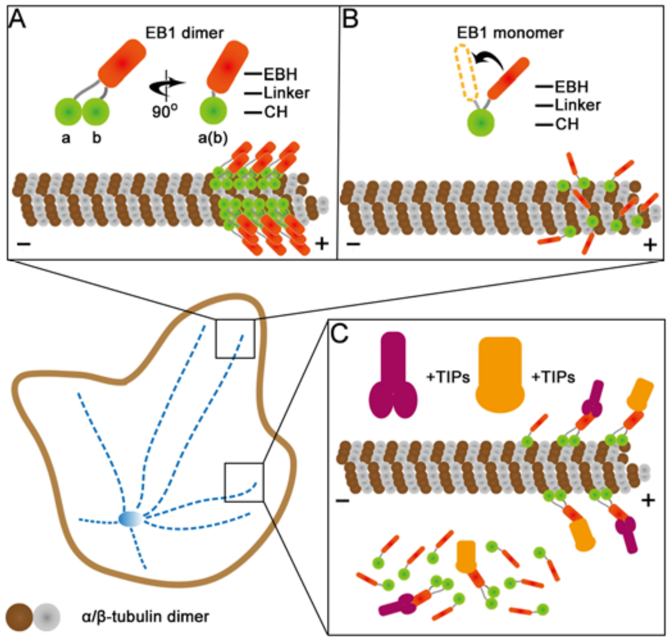

Figure S4. Schematic models showing that EB1 dimerization facilitates its binding to microtubules and other +TIPs. (A) Interaction of EB1 dimers with the microtubule. (B) Interaction of EB1 monomers with the microtubule. (C) Interaction of EB1 dimers and monomers with other +TIPs.
